# Evaluating the Clinical Impact of Metagenomic Next-Generation Sequencing in CNS Infections: A Diagnostic Pathway and Resource Utilization Modeling Study

**DOI:** 10.1093/ofid/ofaf743

**Published:** 2025-12-11

**Authors:** Gerome Vallejos, Carla Kim, Kathryn B Holroyd, Kiran T Thakur

**Affiliations:** Program in Neuroinfectious Diseases, Department of Neurology, Columbia University Irving Medical Center, New York Presbyterian Hospital, New York, New York, USA; Program in Neuroinfectious Diseases, Department of Neurology, Columbia University Irving Medical Center, New York Presbyterian Hospital, New York, New York, USA; Program in Neuroinfectious Diseases, Department of Neurology, Columbia University Irving Medical Center, New York Presbyterian Hospital, New York, New York, USA; Program in Neuroinfectious Diseases, Department of Neurology, Columbia University Irving Medical Center, New York Presbyterian Hospital, New York, New York, USA

**Keywords:** encephalitis, meningitis, metagenomic next-generation sequencing

## Abstract

**Background:**

Diagnosing meningitis and encephalitis remains challenging due to nonspecific clinical presentations and the limitations of traditional microbiological methods. Metagenomic next-generation sequencing (mNGS) offers a broad approach to detect pathogens, but its real-world impact on clinical decision-making remains undefined.

**Methods:**

We used a cohort of patients with confirmed central nervous system infections and autoimmune encephalitis (AE) who underwent traditional microbiological cerebrospinal fluid testing at Columbia University Irving Medical Center. Using published sensitivity and specificity data for mNGS, we applied Bayes’ theorem to calculate different etiology-specific pretest probabilities and model the potential impact in the diagnostic workflows including the number of lumbar punctures (LPs), additional etiologic tests potentially avoided, and time to diagnosis.

**Results:**

The cohort includes 54 patients in the infectious cohort and 29 patients with confirmed autoimmune encephalitis. In a modeled scenario, utilizing an mNGS test, such as Delve Detect, in patients with DNA viral infections (n = 23) could lead to a reduction of up to 88 microbiological tests, 145 days to diagnosis, and 2 LPs in total. For bacterial infections (n = 16), estimated impact included a reduction of 30 microbiological tests, 144 days to diagnosis, and 12 LPs (Table 1). Although fungal, RNA viral and parasitic infections were less common, with adjusted positive predictive values of 92.8%, 89.5%, and 84.6%, respectively. In the autoimmune cohort, a total of 2 LPs, 126 microbiological tests, and 297 days to diagnosis could have been avoided through the use of mNGS.

**Conclusions:**

Our analysis suggests that an mNGS test, such as Delve Detect, could potentially streamline diagnostic and treatment pathways in meningitis and encephalitis of infectious or autoimmune origin.

## Teaser

This study estimates the clinical impact of an mNGS test in meningitis and encephalitis using a Bayesian modeling framework. Findings highlight the potential of mNGS to reduce testing burden and time to diagnosis, ultimately guiding targeted therapy and improving management.

Diagnosing meningitis and encephalitis (ME) remains a clinical challenge due to the wide variety of potential etiologies, both infectious and noninfectious, as well as the confounding, nonspecific presenting symptoms [1]. In the diagnostic workup to identify etiologies, traditional diagnostic methods such as culture, targeted polymerase chain reaction (PCR) assays, and serologic testing are employed; however, they are limited to a narrow list of targeted pathogens, have variable sensitivity, and, at times, are not readily accessible; these factors ultimately lead to delayed results [2]. As a result, many patients with suspected infectious or noninfectious ME are treated with broad, untargeted, empiric therapies while awaiting lengthy and extensive diagnostic testing, yet many of these patients remain without a confirmed diagnosis [3]. Metagenomic next-generation sequencing (mNGS) has emerged as a promising diagnostic tool that enables unbiased detection of pathogens directly from clinical samples, including cerebrospinal fluid (CSF) [4, 5]. By capturing and sequencing nucleic acids from any present organisms, mNGS eliminates the bias of relying on clinical assumptions to identify likely pathogens.

Despite its considerable potential, mNGS is constrained by factors such as high cost, variable turnaround times, variable test performance, and the absence of clinical guidelines to support its implementation in real-world settings [6, 7]. Recent advances have increased access to clinically validated and commercially robust mNGS platforms, such as Delve Detect, that have the potential to increase clinical utility by (1) detecting both RNA and DNA pathogens, thereby increasing diagnostic yield by >20% [8], (2) shortening turnaround times (∼48 hours), and (3) increasing sensitivity and specificity of diagnostic testing [8, 9]. However, its real-world impact on clinical decision-making and resource utilization remains uncertain, particularly when considering integration earlier in clinical diagnostic algorithms. To address this, we developed a probabilistic Bayesian modeling framework to estimate the potential clinical utility of an mNGS test that detects RNA and DNA pathogens in CSF in ∼48 hours.

## METHODS

### Participants

The study utilizes an existing cohort of 111 patients with CNS infection and 85 patients with autoimmune encephalitis (AE) hospitalized at Columbia University Irving Medical Center (CUIMC) between January 1, 2010, and December 31, 2017. The study has institutional review board (IRB) approval from the CUIMC IRB (Protocol AAAR3440), and a waiver of informed consent was obtained for retrospective chart review.

### Data Collection

The infectious cohort was classified by etiology as follows: DNA virus, RNA virus, bacteria, fungus, and parasite. Definitive diagnosis of infectious etiologies was confirmed during the initial hospital stay through detection of a specific pathogen through traditional microbiological methods such as CSF PCR, culture, antibody or antigen testing, or biopsy results. Samples with a confirmed etiology within the first 48-hour window—either by BioFire FilmArray meningitis/encephalitis panel, which returns results within ∼2–4 hours (run in-house at CUIMC), or through CSF PCR, culture, or antibody/antigen testing (also run-in house)—were excluded to isolate the impact of using mNGS on the cohort that did not receive a diagnosis within 48 hours. For the AE cohort, we excluded patients who lacked a definitive etiologic diagnosis, had a diagnosis based on blood tests, or had a known diagnosis upon admission at CUIMC. Cases with inaccessible or incomplete electronic medical records were excluded.

Demographic information, hospitalization details, and diagnostic evaluations were obtained through electronic medical records (EMRs) as previously published [10]. Additional data were collected through chart review, including time from specimen collection to laboratory result reporting, number of lumbar punctures (LPs) performed, range of infectious etiologic tests ordered, and treatment data such as types of drugs administered, dates of initiation, and total number of drugs prescribed during hospitalization.

### Statistical Analysis

We first estimated the frequency of each infectious etiology (bacterial, viral [RNA and DNA], fungal, and parasitic) within the cohort based on confirmed diagnoses. Using these prevalence estimates as prior probabilities, we applied a Bayesian framework to adjust the positive predictive value (PPV) and negative predictive value (NPV) of a hypothetical mNGS test. The mNGS test was modeled to return results within ∼48 hours, reflecting currently available turnaround times, and to detect both RNA and DNA pathogens from CSF, reported by Delve Detect [8]. Test characteristics (sensitivity and specificity) were informed by published performance metrics from existing mNGS studies [8]. Using Bayesian theorem, we calculated post-test probabilities—adjusted PPV and NPV—for each pathogen category [11]. To estimate the clinical impact of mNGS, we first calculated the average number of LPs, infectious etiologic tests, and days to diagnosis in the standard diagnostic pathway.

Using the adjusted PPV in the infectious cohort, we estimated the number of true positives that would have been identified had mNGS been applied at presentation. For these hypothetical true-positive cases, we assigned a simplified diagnostic scenario consistent with using mNGS early in the diagnostic workup: 1 LP, 1 etiologic test, and 2 days to diagnose per patient. We then calculated the difference between the scenario of using mNGS early and the observed average time to diagnosis, the number of LPs avoided, microbiological tests avoided, and diagnostic days saved.

Using the adjusted NPVs in the autoimmune cohort, we estimated the number of true negatives that would have been identified had mNGS been applied at presentation. For these hypothetical true-negative cases, we assigned a simplified diagnostic pathway consistent with the use of mNGS early: 1 LP, 0 additional etiologic tests, and 2 days to diagnose per patient, assuming that a negative result would have precluded further microbiologic workup. We then calculated the difference between this idealized mNGS-negative scenario and the observed averages to estimate the number of LPs, microbiological tests, and days to infection rule-out saved. All modeling and analyses were conducted using R, version 4.3.2.

## RESULTS

**Figure 1.**
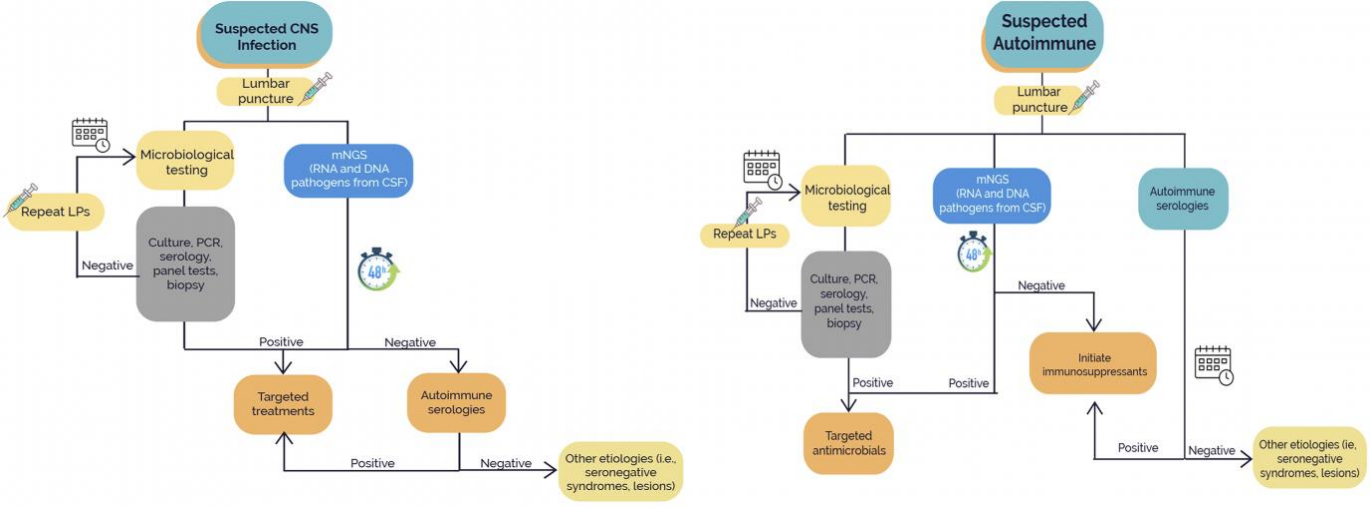
Decision tree representing etiologic testing for suspected CNS infections. Other potential causes—including metabolic, structural, and toxic etiologies—were excluded based on clinical assessment, laboratory testing, and neuroimaging. Abbreviations: CNS, central nervous system; CSF, cerebrospinal fluid; mNGS, metagenomic next-generation sequencing; PCR, polymerase chain reaction.

We developed a decision tree model to represent the diagnostic workup for patients with suspected CNS infections and suspected autoimmune encephalitis (Figure 1), comparing 2 hypothetical scenarios: standard-of-care microbiological testing vs mNGS early in the workup. In both arms, a positive result prompted immediate targeted treatment, while negative results led to additional investigations, including autoimmune and tertiary testing.

**Figure 2.**
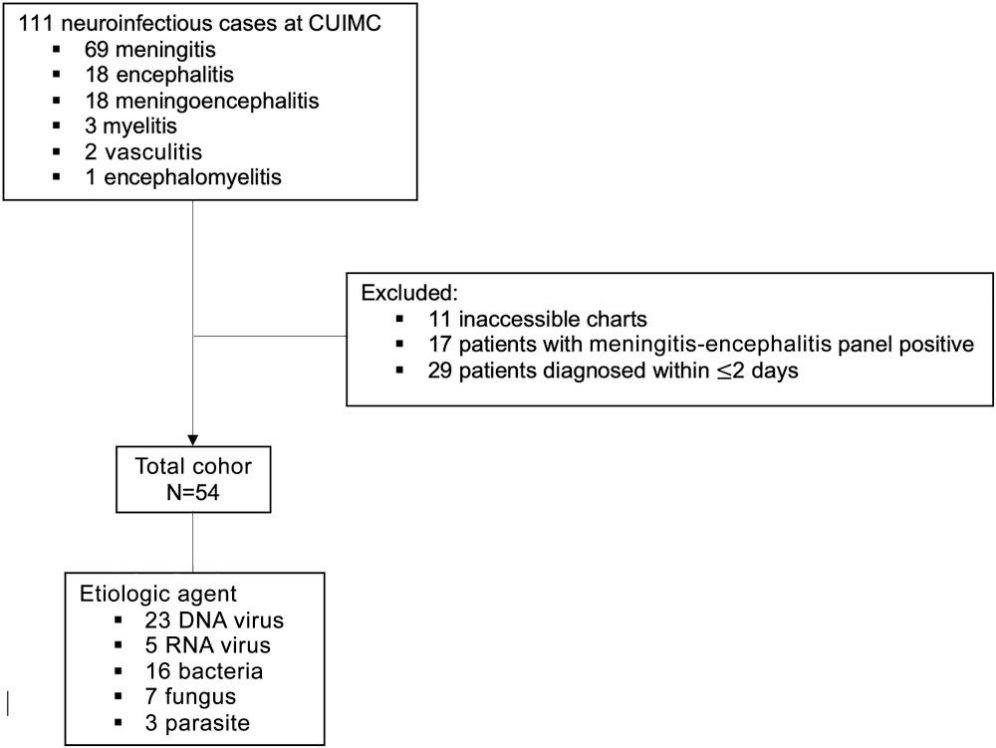
Infectious cohort creation. Abbreviation: CUIMC, Columbia University Irving Medical Center.

A total of 54 patients in the infectious cohort were divided into 4 category-specific etiologies: 23 DNA viruses, 5 RNA viruses, 16 bacterial, 7 fungal, and 3 parasitic (Figure 2). Patients diagnosed with DNA viruses (n = 23) underwent a total of 27 LPs (1.1 per patient) and 132 etiologic tests (5.7 per patient), with an average time to diagnosis of 8.6 days (Supplementary Table 1). Those with RNA viruses (n = 5) had 6 LPs (1.2 per patient), 9 tests (1.8 per patient), and the shortest average time to diagnosis, at 4.8 days. Bacterial infections (n = 16) required 29 LPs (1.8 per patient) and 51 tests (3.2 per patient), with a longer average time to diagnosis of 11.6 days. Fungal cases (n = 7) involved 11 LPs (1.5 per patient) and 41 tests (5.8 per patient), with the longest diagnostic delay at 12.3 days. Parasite-related cases (n = 3) included 3 LPs (1.0 per patient), 16 tests (5.3 per patient), and an average time to diagnosis of 6.6 days.

Modeling projections indicated that earlier implementation of mNGS tests that detect both RNA and DNA pathogens within 48 hours, such as Delve Detect, may reduce diagnostic procedures and time to diagnosis across several infectious etiologies. In DNA viral infections, the model estimated a possible decrease of 88 microbiological tests, 145 days to diagnosis, and 2 lumbar punctures (LPs). Bacterial cases showed a projected reduction of 30 tests, 144 days to diagnosis (inpatient), and 12 LPs. In fungal infections, with an adjusted positive predictive value (PPV) of 92.8%, estimated reductions included 29 tests, 61 days to diagnosis, and 3 LPs. Though RNA viral and parasitic infections were less frequent, their respective PPVs of 89.5% and 84.6% suggest that earlier mNGS use may reduce the frequency and number of tests and ultimately minimize days to diagnosis (Table 1).

**Table 1.** Impact of Adjusted mNGS PPV on Diagnostic Efficiency and Resource Use by Pathogen Type

	Adjusted mNGS PPV	Total No. of Patients	Total No. of Patients who Will Test Positive With mNGS	Total No. of LPs Avoided	Total No. of Etiologic Tests Avoided	Total No. of Days Saved
DNA virus	0.984	23	∼ 22	2	88	145
RNA virus	0.895	5	∼ 4	0	4	11
Bacteria	0.974	16	∼ 15	12	30	144
Fungus	0.928	7	∼ 6	3	29	61
Parasite	0.846	3	∼ 2	0	9	9

Abbreviations: LPs, lumbar punctures; mNGS, metagenomic next-generation sequencing; PPV, positive predictive value.

**Figure 3.**
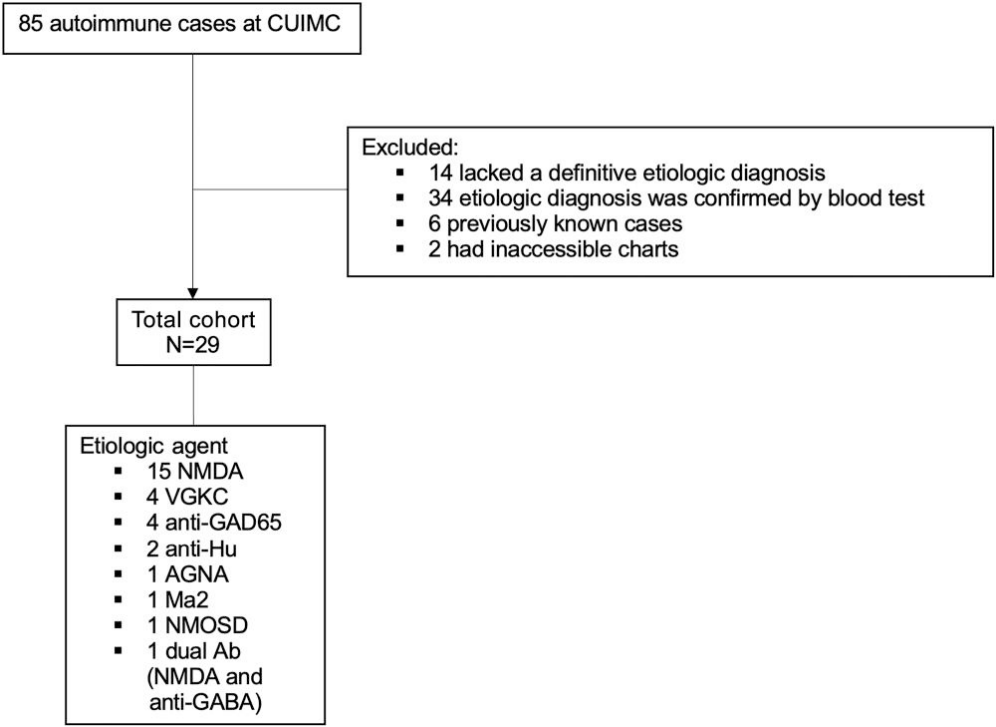
Autoimmune cohort creation. Abbreviation: CUIMC, Columbia University Irving Medical Center.

A total of 29 patients with confirmed autoimmune etiologies were included in the study (Figure 3). Among these individuals, 33 LPs were performed, with an average of 1.1 per patient. In total, 137 microbiological tests were conducted, with a mean of 4.7 tests per patient (Supplementary Table 2). Based on modeling assumptions, if mNGS had been used early in the diagnostic process, ∼27 of these patients may have tested true negative, which could have been associated with avoidance of up to 2 LPs, 126 microbiological tests, and 297 days to infection rule-out saved (Table 2).

**Table 2.** Impact of Adjusted mNGS NPV on Diagnostic Efficiency and Resource Use by Pathogen Type

	Adjusted mNGS NPV	Total No. of Patients	Total No. of Patients who Will Test Negative With mNGS	Total No. of LPs Avoided	Total No. of Etiologic Tests Avoided	Days to Infection Rule-Out Saved
Autoimmune cases	0.984	29	∼ 27	2	126	297

Abbreviations: LPs, lumbar punctures; mNGS, metagenomic next-generation sequencing; NPV, negative predictive value.

## DISCUSSION

Because of its ability to detect a wide array of potential pathogens early in the course of disease, mNGS has emerged as a potential tool to simplify the diagnosis of ME and reduce the need for multiple tests and procedures. In this study, we utilized a Bayesian modeling approach to evaluate the potential utility of mNGS in clinical practice and highlight its potential utility in minimizing diagnostic steps and delays to treatment commonly encountered in the evaluation of ME patients. Our findings suggest that many of these procedures and prolonged evaluations could potentially be avoided. With its comprehensive pathogen detection and high positive and negative predictive values, mNGS offers the ability to deliver more definitive diagnoses in cases where traditional methods are inconclusive or too narrowly focused [12, 13].

Prior studies have highlighted the potential of mNGS to improve diagnosis of CNS infections, particularly detecting pathogens where conventional diagnostics have failed [4, 8, 9]. Large multicenter studies have demonstrated that mNGS identifies causative pathogens in ∼21.8%–77% of CNS infection cases, making the diagnosis in a significant proportion of patients where traditional culture, PCR, and serological testing have failed [8, 14, 15]. For instance, in a recent 7-year cohort study [8], mNGS correctly identified infectious causes in 63.1% of CNS infections—substantially outperforming indirect serologic (28.8%) and standard direct detection from CSF (45.9%). When analysis was limited to cases where CSF direct pathogen detection was applicable, mNGS sensitivity rose to 86%. Other recent analyses report pooled sensitivity around 70%–77% and specificity of 93%–96% for CNS infections [14, 15]. These data highlight the added value of mNGS as part of an integrated, multidimensional diagnostic process for complex CNS cases. Additionally, mNGS of non-CSF samples has identified rare pathogens that were difficult to detect using conventional methods. In 1 representative large cohort, the detection rate was 78.9% with mNGS vs 20% with conventional testing in bronchoalveolar lavage fluid samples [16]. Another analysis found that detection rates improved from 22.7% (conventional) to 70.7%–85.2% (mNGS) in lower respiratory tract infections [17]. mNGS has thereby offered not only speed and diagnostic breadth, but also influenced clinical decision-making in critical care settings.

Beyond infectious causes, another important emerging application of mNGS as a tool is to shorten the time to diagnosis of autoimmune diseases of the CNS. Our findings suggest that LPs and additional microbiological tests may also have been able to be avoided if a negative mNGS result had been available earlier. In the AE cohort, >20 different pathogens were explored (Supplementary Table 3), ranging from more common pathogens such as HSV 1/2 to less common ones such as *Tropheryma whipplei* and *Borrelia* (Lyme disease). In these diagnostically ambiguous situations, a negative result by mNGS can help reduce the likelihood of an infectious cause, enabling clinicians to pursue other etiologies. Additionally, a negative mNGS result could potentially safely de-escalate antimicrobial therapies and pivot toward autoimmune or inflammatory treatments. In the cohort of infectious ME, a total of 259 antimicrobials were prescribed (Supplementary Tables 4 and 5) before the etiological diagnosis, while in the AE cohort, 9 different antimicrobials were prescribed before diagnosis of AE (Supplementary Table 6). While real-world clinical decision-making differs from hypothetical modeling—often requiring expert input and the nuanced interpretation of multiple diagnostic modalities—the findings of this study should not be interpreted in isolation [18]. Rather, they suggest that mNGS has potential value when integrated with conventional microbiological testing, particularly in diagnostically challenging cases where establishing an etiology is prolonged or complex. It is most effective as part of a comprehensive diagnostic strategy rather than as a standalone test.

While this modeling study presents valuable insights, it is important to highlight that this work is based on a theoretical model built from previously published performance metrics. As such, the results are intended to illustrate theoretical benefits rather than real outcomes. The model also makes several simplifying assumptions, including consistent diagnostic behavior and optimal test performance, which does not fully reflect the variability encountered in real-world health care settings. In practice, mNGS performance can vary depending on sample quality, sequencing depth, background contamination, and interpretation thresholds. Additionally, the microbiological testing was assumed to have diagnosed suspected infections with 100% PPV—published studies have shown that mNGS in CSF has led to an increase in diagnostic yield of over 20% [8]—and this model does not address the potential added diagnostic yield of mNGS when diagnosing ME.

Furthermore, the performance of mNGS tests varies greatly between manufacturers—Delve Detect covers both RNA and DNA pathogens; however, other commercially available mNGS tests may only detect other analytes such as cell-free DNA or target different sample types (ie, BAL or plasma), and sensitivity and specificity vary across pathogen types, particularly those in low abundance. Given the variability in test performance, further evaluation of each platform is needed to fully investigate the use of mNGS in diagnosing noninfectious mimics of CNS infection such as autoimmune or paraneoplastic syndromes. Additionally, our model assumes that mNGS results could directly guide clinical decision-making, but this may not always be the case. Clinicians may still pursue additional testing based on clinical presentation, pretest probability, concerns about false positives and incidental findings, or individual intrinsic decision-making heuristics. Lastly, the current model was constructed as an idealized framework to evaluate potential diagnostic and resource outcomes under controlled assumptions. Incorporating cases that remain undiagnosed even after mNGS will be important to more accurately reflect real-world practice. Consequently, the estimates of LPs, additional tests, and time to diagnosis reflect an idealized scenario and may overstate the real-world impact of mNGS.

In summary, while our data indicate that mNGS could potentially be used to complement existing diagnostic workups in the evaluation of ME, particularly when used early in the diagnostic process, these findings are exploratory and based on modeled projections. Future prospective studies that assess real-world diagnostic impact, clinical outcomes, and cost-effectiveness will be essential to determine how and when mNGS should be incorporated into standard diagnostic algorithms.

## Supplementary Material

ofaf743_Supplementary_Data

## Supplementary Data

Supplementary materials are available at *Open Forum Infectious Diseases* online. Consisting of data provided by the authors to benefit the reader, the posted materials are not copyedited and are the sole responsibility of the authors, so questions or comments should be addressed to the corresponding author.
