# Supplementary material for: Evaluating the Clinical Impact of Metagenomic Next-Generation Sequencing in CNS Infections: A Diagnostic Pathway and Resource Utilization Modeling Study
Source: Open Forum Infect Dis. 2025 Dec 11;13(1):ofaf743. doi: 10.1093/ofid/ofaf743 (PMC12750319; doi:10.1093/ofid/ofaf743)
Supplement: ofaf743_Supplementary_Data [file ofaf743_supplementary_data.docx]

**Supplementary**

Table 1 Summary of Lumbar Punctures, Etiologic Tests, and Time to Diagnosis in the Infectious Cohort

|  | Total number of patients | Total number of LP done | Average LP/patient | Total number of Etiologic Tests done | Average Etiologic test/patient | Total number of Days to Diagnosis | Average Days to Diagnosis/patient |
| --- | --- | --- | --- | --- | --- | --- | --- |
| DNA Virus | 23 | 27 | 1.1 | 132 | 5.7 | 199 | 8.6 |
| RNA Virus | 5 | 6 | 1.2 | 9 | 1.8 | 24 | 4.8 |
| Bacteria | 16 | 29 | 1.8 | 51 | 3.2 | 186 | 11.6 |
| Fungus | 7 | 11 | 1.5 | 41 | 5.8 | 86 | 12.3 |
| Parasite | 3 | 3 | 1 | 16 | 5.3 | 20 | 6.6 |

Table 2 Summary of Lumbar Punctures and Etiologic Tests in the Autoimmune Cohort

|  | Total number of patients | Total number of LP done | Average LP/patient | Total number of Etiologic Tests done | Average Etiologic test/patient | Total Number of Days to Diagnosis | Average days to diagnosis/patient |
| --- | --- | --- | --- | --- | --- | --- | --- |
| Autoimmune cases | 29 | 33 | 1.1 | 137 | 4.7 | 384 | 13 |

Table 3 List of Pathogens tested prior to AE diagnosis

| Pathogens |
| --- |
| HSV 1 & 2 |
| CMV |
| EBV |
| VZV |
| HHV-6 |
| Enterovirus |
| Adenovirus |
| JCV (JC Virus) |
| Parvovirus B19 |
| HTLV |
| West Nile Virus (WNV) |
| Mycoplasma |
| Legionella |
| Bartonella |
| Borrelia (Lyme Disease) |
| Toxoplasma |
| Treponema pallidum (Syphilis) |
| Cryptococcus (CRAG/Ag) |
| Coccidioides |
| Histoplasma |
| Acid-Fast Bacilli (AFB, TB) |
| Tropheryma whipplei |
| Fungal culture/general fungal |
| Bacterial culture/CSF culture |
| NYS Encephalitis Panel |
| Meningitis/Encephalitis Panel |
| Fungitell |

Table 4 Summary of Antimicrobials in the Infectious Cohort

|  | Total number of patients | Total number antimicrobials given |
| --- | --- | --- |
| Infectious cohort | 54 | 259 |

Table 5 Antimicrobials in the Infectious Cohort Prescribed Prior to Etiologic Diagnosis

| Treatment | Count |
| --- | --- |
| Vancomycin | 42 |
| Ceftriaxone | 39 |
| Acyclovir | 28 |
| Piperacillin-Tazobactam | 12 |
| Trimethoprim- Sulfamethoxazole | 12 |
| Fluconazole | 13 |
| Amphotericin B | 9 |
| Cefepime | 8 |
| Ampicillin | 11 |
| Azithromycin | 11 |
| Linezolid | 6 |
| Nystatin | 6 |
| Pyrimethamine | 6 |
| Penicillin G | 6 |
| Clindamycin | 5 |
| Valacyclovir | 5 |
| Atovaquone | 4 |
| Ritonavir | 4 |
| Levofloxacin | 3 |
| Voriconazole | 3 |
| Micafungin | 3 |
| Atazanavir | 3 |
| Rifampin | 3 |
| Cefazolin | 2 |
| Darunavir | 2 |
| Flucytosine | 2 |
| Clotrimazole | 1 |
| Miconazole | 1 |
| Dapsone | 1 |
| Ketoconazole | 1 |
| Econazole | 1 |
| Aztreonam | 1 |
| Emtricitabine-Tenofovir | 1 |
| Efavirenz-Emtricitabine-Tenofovir | 1 |
| Isoniazid | 1 |
| Ethambutol | 1 |
| Pyrazinamide | 1 |

Table 6 List of Antimicrobials given prior to AE diagnosis

| Antibiotic | Frequency | Percent of regimens (%) |
| --- | --- | --- |
| Acyclovir | 7/10 | 70.0% |
| Vancomycin | 6/10 | 60.0% |
| Pip-Taz | 3/10 | 30.0% |
| Ceftriaxone | 2/10 | 20.0% |
| TMP-SMX | 2/10 | 20.0% |
| Linezolid | 1/10 | 10.0% |
| Metronidazole | 1/10 | 10.0% |
| Fluconazole | 1/10 | 10.0% |
| Oxacillin | 1/10 | 10.0% |
